# Analysis of Optimal Sequential State Discrimination for Linearly Independent Pure Quantum States

**DOI:** 10.1038/s41598-018-24575-w

**Published:** 2018-04-25

**Authors:** Min Namkung, Younghun Kwon

**Affiliations:** Department of Applied Physics, Hanyang University, Ansan, Kyounggi-Do 425-791 South Korea

## Abstract

Recently, J. A. Bergou *et al*. proposed sequential state discrimination as a new quantum state discrimination scheme. In the scheme, by the successful sequential discrimination of a qubit state, receivers Bob and Charlie can share the information of the qubit prepared by a sender Alice. A merit of the scheme is that a quantum channel is established between Bob and Charlie, but a classical communication is not allowed. In this report, we present a method for extending the original sequential state discrimination of two qubit states to a scheme of *N* linearly independent pure quantum states. Specifically, we obtain the conditions for the sequential state discrimination of *N* = 3 pure quantum states. We can analytically provide conditions when there is a special symmetry among *N* = 3 linearly independent pure quantum states. Additionally, we show that the scenario proposed in this study can be applied to quantum key distribution. Furthermore, we show that the sequential state discrimination of three qutrit states performs better than the strategy of probabilistic quantum cloning.

## Introduction

Quantum state discrimination is one of the main research topics in quantum information processing. The information used in quantum communication or quantum computing is encoded by quantum states. Naturally, this requires quantum state discrimination. In quantum state discrimination, a sender Alice prepares a quantum state with a prior probability and sends it to a receiver Bob. Bob, knowing the prior probability, performs a measurement to discriminate the quantum state of Alice. When Alice’s quantum states are orthogonal to each other, Bob can always discriminate Alice’s quantum states. However, Alice’s quantum states are usually not orthogonal. Therefore, Bob cannot perfectly discriminate these quantum states^1^. The result of Bob’s measurement can be divided into two cases:conclusive results and inconclusive results. When Bob obtains an inconclusive result, he cannot find any information about Alice’s quantum state. When Bob’s measurement result is conclusive, Bob can obtain information about Alice’s quantum state. Minimum error discrimination(ME)^1–5^ is the strategy for discriminating quantum states with measurements, which always provide conclusive results with a minimum error. When the probability of incorrect guessing becomes zero, a strategy called unambiguous discrimination(UD) is used. Therefore, in the unambiguous discrimination(UD)^6–9^, measurements are designed to provide conclusive results which are always correct, and inconclusive result which is minimized. UD can be applied only when Alice’s quantum states are either linearly independent pure states^10^ or mixed quantum states whose support space does not completely overlap^11^. Other strategies for quantum state discrimination employ maximal confidence(MC)^12^, error margin(EM) methods^13–16^, and fixed rate of inconclusive result(FRIR) methods^17–20^.

Recently, Bergou *et al*.^21^ proposed *sequential state discrimination(SSD)*, a new strategy for quantum state discrimination, that contains multi-receivers. Suppose that the receivers are Bob and Charlie. The receivers knows the prior probability of quantum states, prepared by Alice. In this strategy, Bob discriminates Alice’s quantum state using nonoptimal unambiguous discrimination. When Bob obtain a conclusive result, he sends his post-measurement state to Charlie. Charlie performs an optimal unambiguous discrimination on Bob’s post-measurement state. In this strategy, there is no classical communication between Bob and Charlie. If Bob and Charlie can successfully discriminate Alice’s quantum state, every party can share the information of Alice’s quantum state. In fact, sequential state discrimination strategy originates from the question of “Can one obtain the information of original quantum sate by performing a measurement on the post-measurement state?”^22^. The optimal success probability of sequential state discrimination for two pure qubits with identical prior probabilities was analytically provided by Bergou *et al*.^21,23^, which was shown in experiment^24^. A solution to sequential state discrimination for two pure qubits with arbitrary prior probabilities was suggested by Zhang *et al*.^25^. Hillery *et al.*^26^ proposed sequential state discrimination for a symmetric *N*−qudit, which can be obtained by embedding qubits into an extra Hilbert space. In addition, the authors of present report provided the sequential state discrimination for mixed qubit states^27^.

In this report, we investigate the structure of sequential state discrimination for *N* pure qudit states. When *N* pure states are linearly independent, a Gram matrix composed of pure states is positive-definite, which implies the existence of the inverse of the Gram matrix^28^. From the Gram matrix, the POVM of Bob and Charlie for unambiguous discrimination may be found^29,30^. In general, the POVM is composed of *N* + 1 complex positive *D*-dimensional matrices. However, in this report, we will let the POVM correspond to a vector composed of *N* real numbers. The detailed procedure will be explained in the formulation of problem. The POVM set, used in unambiguous discrimination, is convex^30^, and the set of real vectors corresponding to it is convex. When *N* = 2, the convex set can be easily understood. However, when *N* ≥ 3, the convex set has not been investigated.

In this report, we study the structure of the convex set of linearly independent *N* pure states for sequential state discrimination, mainly dealing with the convex set of *N* = 3^31–33^. Because Bob performs an nonoptimal unambiguous discrimination, the real vector corresponding to Bob’s POVM exists inside the convex set. However, Charlie performs the optimal unambiguous discrimination, and the real vector corresponding to Charlie’s POVM exists on the surface of the convex set. Charlie’s convex set depends on Bob’s POVM. When Bob uses an optimal unambiguous discrimination, the Charlie’s convex set element is only a zero vector. Meanwhile, when Bob performs a nonoptimal unambiguous discrimination, the Charlie’s convex set contains other elements, except for the zero vector. This implies that Charlie can obtain some information from the post-measurement states of Bob.

## Results

Now, let us propose a sequential state discrimination for *N* linearly independent pure quantum states. Up to now, the solution to sequential state discrimination for two linearly independent pure qubit states is known, which is the result of Bergou *et al*.^21,25^. Therefore, we need to formulate the sequential state discrimination for *N* linearly independent pure quantum states, which can be considered as a optimization problem based on the conditions of POVM of receivers Bob and Charlie. Then, we obtain a solution for the cases of *N* = 3 linearly independent quantum states with arbitrary prior probabilities. In addition, we compare our strategy with probabilistic quantum cloning strategy. We find that sequential state discrimination for *N* = 3 linearly independent quantum states performs better than the strategy of probabilistic quantum cloning. Finally, we show that our proposal can be used for quantum key distribution.

### Scenario of Sequential State Discrimination

Let us explain a sequential state discrimination scenario of *N* linearly independent pure quantum states. A sender Alice prepares a quantum state |*ψ*_*i*_〉 with a prior probability *q*_*i*_, out of a set of *N* linearly independent pure quantum states {|*ψ*_1_〉, |*ψ*_2_〉, …, |*ψ*_*N*_〉}. Then, she sends this quantum state to Bob. Bob discriminates Alice’s quantum state, using POVM {*M*_0_, *M*_1_, …, *M*_*N*_}. Here, ${M}_{i}(i\ne 0)$ is an element corresponding to conclusive result *i* ∈ {1, …, *N*} and *M*_0_ is an element corresponding to inconclusive result *i* = 0. If Bob obtains *i* ∈ {1, …, *N*} as a measurement result, he sends his post-measurement state $|{\psi ^{\prime} }_{i}\rangle$ to Charlie. Then, Charlie performs POVM $\{{M^{\prime} }_{0},{M^{\prime} }_{1},\cdots ,{M^{\prime} }_{N}\}$ on Bob’s post-measurement state, to determine the post-measurement state of Bob. Likewise, Charlie’s ${M^{\prime} }_{i}(i\ne 0)$ corresponds to conclusive result *i* ∈ {1, …, *N*} and ${M^{\prime} }_{0}$ corresponds to inconclusive result *i* = 0. Receivers Bob and Charlie should obey the following rules.

*Rule 1*. Bob discriminates Alice’s quantum state using a nonoptimal unambiguous discrimination strategy. However, using an optimal unambiguous discrimination strategy, Charlie mesures Bob’s post-measurement state.

*Rule* 2. Classical communication is not allowed between Bob and Charlie. Therefore, the only way for Charlie to obtain information about Alice’s quantum state is to measure Bob’s post-measurement state.

Therefore, the probability that Bob and Charlie successfully share Alice’s quantum state is given by

1 $${P}_{s}^{(B,C)}=\sum _{i=1}^{N}{q}_{i}\langle {\psi }_{i}|{M}_{i}|{\psi }_{i}\rangle \langle {\psi ^{\prime}}_{i}|{M^{\prime} }_{i}|{\psi ^{\prime} }_{i}\rangle .$$

The important problem in sequential state discrimination is in finding the POVM condition of Bob and Charlie, for optimizing the success probability of Eq. (1). If the optimal value of (1) is not zero, Bob and Charlie have a chance of successfully sharing Alice’s quantum state. When Alice prepares a qubit state out of two pure qubit states, the success probability is proven to be nonzero^21,23–25^. To date, researchers have considered the optimal success probability only for two qubit states. In this report, proposing the sequential state discrimination for *N* linearly independent pure quantum states, we provide a method for finding the optimized success probability condition.

### Formulation of Optimization Problem

Now, let us describe how to formulate an optimization problem for the sequential state discrimination of *N* linearly independent pure quantum states. The conditions for Bob to discriminate Alice’s quantum state without error are (B1) *M*_*i*_ ≥ 0(∀*i* ∈ {0, …, *N*}), (B2) *M*_0_ + *M*_1_ + … + *M*_*N*_ = *I*, (B3) $\langle {\psi }_{j}|{M}_{i}|{\psi }_{j}\rangle =\mathrm{0(}\forall i\ne j)$. Let us assume that $G={\{\langle {\psi }_{i}|{\psi }_{j}\rangle \}}_{i,j=1}^{N}$ is a Gram matrix made by Alice’s pure states. When Alice’s pure states are linearly independent, *G* > 0(↔ ∃*G*^−1^)^28^ is satisfied. Therefore, the POVM that can discriminate every Alice’s pure state without error can be described by^30^,

2 $${M}_{i}={\alpha }_{i}|{\tilde{\psi }}_{i}\rangle \langle {\tilde{\psi }}_{i}|,|{\tilde{\psi }}_{i}\rangle =\sum _{j=1}^{N}{G}_{ji}^{-1}|{\psi }_{j}\rangle .$$

where 0 ≤ *α*_*i*_ ≤ 1, *i* ∈ {1, …, *N*}. If *M*_0_ = *I* − (*M*_1_ + *M*_2_ + … + *M*_*N*_) is non-negative, (B1) and (B2) also hold. Eq. (2) implies that Bob’s POVM can correspond to a real vector (*α*_1_, *α*_2_, …, *α*_*N*_) in *N*−dimensional vector space. Additionally, Bob’s POVM set is convex^30^. The convex set is denoted as $C\subset {{\mathbb{R}}}^{N}$. The conditions under which *M*_0_ is positive-semidefinite are equivalent to 〈*ψ*|*M*_0_|*ψ*〉 ≥ 0 for an *N* dimensional vector |*ψ*〉^28^. Because |*ψ*〉 can be obtained from a linear combination of {|*ψ*_1_〉, …, |*ψ*_*N*_〉}, *C* is an *N* dimensional real vector space where $\tilde{G}={\{\langle {\psi }_{i}|{M}_{0}|{\psi }_{j}\rangle \}}_{i,j=1}^{N}$ is non-negative. Every component of real vector *α*_*i*_, which is an element of *C*, is non-negative. Then, we have the following conjecture about *C*:

#### **Conjecture 1**.

*C, being the set of N dimensional real vectors, is a convex set composed of N* + 1 *vertices. The edges of convex set C contain N segments satisfying α*_*i*_ = 0.

In Conjecture 1, convex set *C* contains a line segment *α*_*i*_ = 0, since there exists a case where Bob discriminates unambiguously remaining quantum states except *i*-th pure state. For *N* = 3, the geometric argument can be found in refs^31–33^. Let us assume that Bob’s set of post-measurement states, which corresponds to conclusive result of Bob, is $\{|{\psi ^{\prime}}_{1}\rangle ,\cdots ,|{\psi ^{\prime}}_{N}\rangle \}$. When Bob discriminates Alice’s quantum state using a nonoptimal unambiguous discrimination, information remains in Bob’s post measurement state, which can be obtained by Charlie. Additionally, Bob’s post-measurement states are linearly independent, which implies that Charlie’s POVM can discriminate Bob’s post-measurement state without error. The conditions for Charlie’s POVM, which can discriminates the Bob’s post-measurement state without error, are (C1) ${M^{\prime} }_{i}\ge \mathrm{0(}\,\forall \,i\,\in \,\mathrm{\{0,}\cdots ,N\})$, (C2) ${M^{\prime} }_{1}+{M^{\prime} }_{2}+\cdots +{M^{\prime} }_{N}=I$, (C3) $\langle {\psi ^{\prime} }_{j}|{M^{\prime} }_{i}|{\psi ^{\prime} }_{j}\rangle =0(\,\forall \,i\ne j)$. When Bob’s post-measurement states are linearly independent, if their Gram matrix is $G^{\prime} ={\{\langle {\psi ^{\prime} }_{i}|{\psi ^{\prime} }_{j}\rangle \}}_{i,j=1}^{N}$, we have *G*′ > 0(↔∃*G*′^−1^). Then, Charlie’s POVM becomes

3 $${M^{\prime} }_{i}={\alpha ^{\prime} }_{i}|{\tilde{\psi }^{\prime} }_{i}\rangle \langle {\tilde{\psi }^{\prime} }_{i}|,|{\tilde{\psi }^{\prime} }_{i}\rangle =\sum _{j=1}^{N}{G^{\prime} }_{ji}^{-1}|{\psi ^{\prime} }_{j}\rangle .$$

where 0 ≤ *α*_*i*′_ ≤ 1, *i* ∈ {1, …, *N*}. If ${M^{\prime} }_{0}=I-({M^{\prime} }_{1}+{M^{\prime} }_{2}+\cdots +{M^{\prime} }_{N})$ is non-negative, (C1) and (C2) also hold. Then, Charlie’s POVM corresponds to the *N* dimensional real vector $({\alpha ^{\prime} }_{1},{\alpha ^{\prime} }_{2},\cdots ,{\alpha ^{\prime} }_{N})$. Let the set of the real vectors be $C^{\prime} \subset {{\mathbb{R}}}^{N}$. Like in the case of Bob, *C*′ is the set of *N* dimensional real vectors, where $\tilde{G}^{\prime} ={\{\langle {\psi ^{\prime} }_{i}|{M^{\prime} }_{0}|{\psi ^{\prime} }_{j}\rangle \}}_{i,j=1}^{N}$ is non-negative. The set is convex and depends on Bob’s POVM. Then, we have the following Conjecture about *C*′.

#### **Conjecture 2**.

*When Bob performs an nonoptimal unambiguous discrimination, C*′ *has a non-zero element. C*′ *depends on Bob’s POVM and is composed of N* + 1 *vertices. The edges of C*′ *contain N segments fulfilling* ${\alpha ^{\prime} }_{i}=0$.

**Figure 1:**
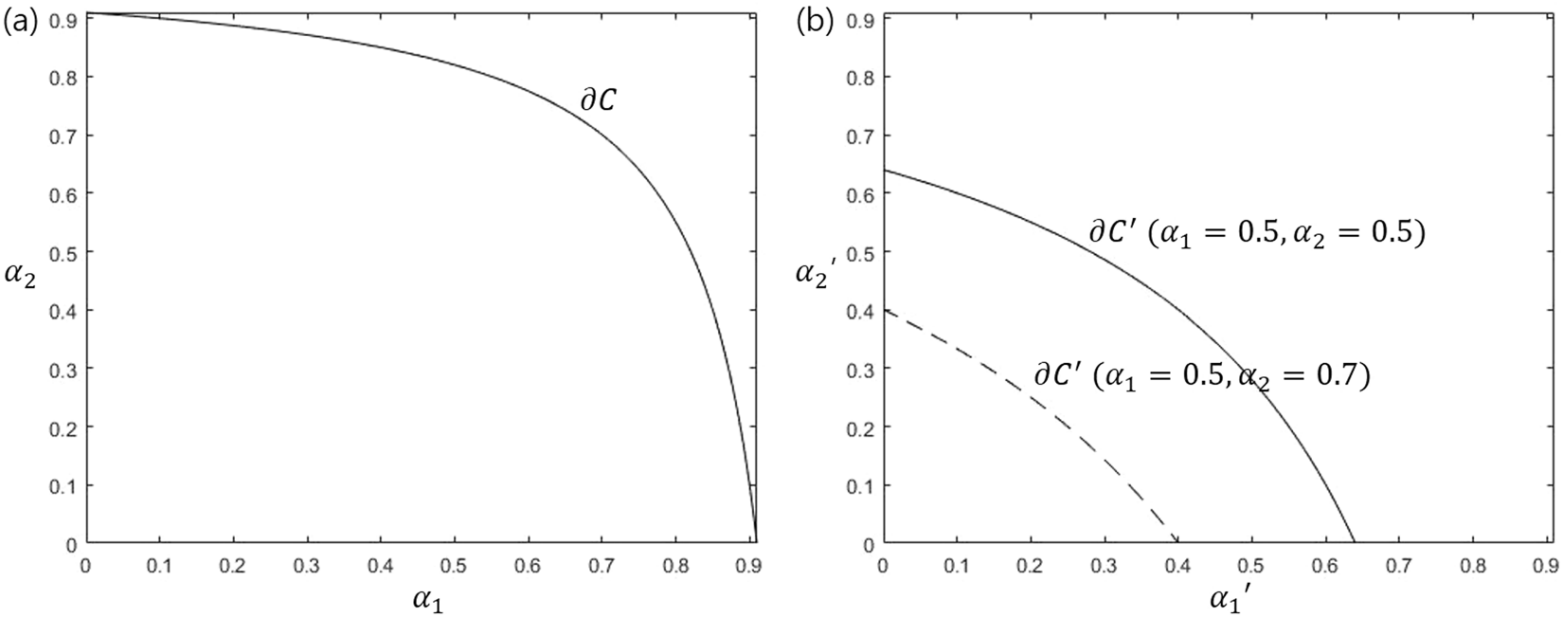
In sequential state discrimination of two pure qubits, the convex sets of Bob and Charlie, *C* and *C*′. (**a**) shows the Bob’s convex set *C* when the overlap between two pure qubits is *s* = 0.3. In (**b**), solid(dashed) line displays the boundary ∂*C*′ of Charlie’s convex set when (*α*_1_, *α*_2_) = (0.5, 0.5)((*α*_1_, *α*_2_) = (0.5, 0.7)).

**Figure 2:**
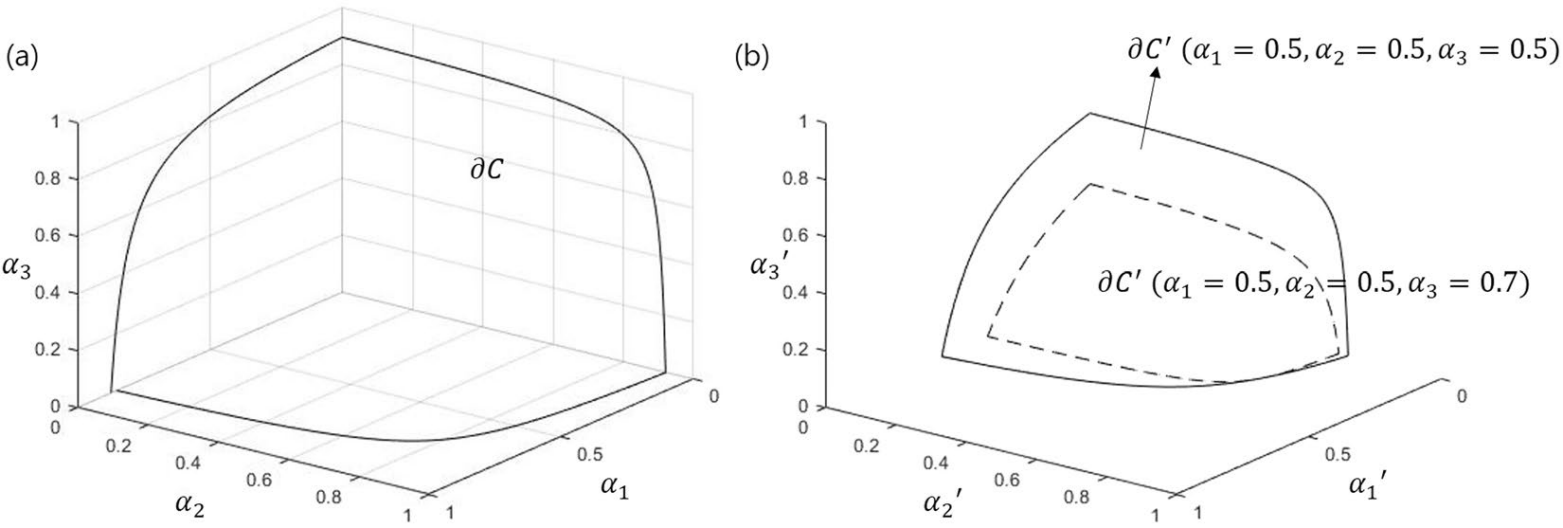
Two set *C* and ∂*C*′ that contain the real vectors (*α*_1_, *α*_2_, *α*_3_) and $({\alpha ^{\prime} }_{1},{\alpha ^{\prime} }_{2},{\alpha ^{\prime} }_{3})$ corresponding to the POVM of Bob and Charlie. Here, ∂*C*′ is a surface of the convex set *C*′. Here, we assume that the overlap among three pure qutrits are *s*_1_ = 0.2, *s*_2_ = 0.3, and *s*_3_ = 0.25. In (**a**), it displays the Bob’s convex set *C*. In (**b**), solid line(dashed line) shows the boundary of Charlie’s convex set *C*′ when *α*_1_ = *α*_2_ = *α*_3_ = 0.5 (*α*_1_ = *α*_2_ = 0.5, *α*_3_ = 0.7)

In Conjecture 2, the fact that convex set *C*′ contains a line segment of ${\alpha ^{\prime} }_{i}=0$ means that there exists a case where Charlie discriminates unambiguously remaining quantum states except *i*-th state. It can be shown from Figs 1 and 2 that Conjecture 2 is satisfied when *N* = 2, 3. In the following section, we will explain how Bob’s POVM affects *C*′. In sequential state discrimination, Bob performs a nonoptimal unambiguous discrimination, and (*α*_1_, …, *α*_*N*_) belongs to the interior of *C*, int(*C*). Meanwhile, Charlie performs an optimal unambiguous discrimination, and a vector $({\alpha ^{\prime} }_{1},\cdots ,{\alpha ^{\prime} }_{N})$ $(\,\forall \,{\alpha ^{\prime} }_{i}\ne 0)$ is located in the boundary of *C*′, ∂*C*′. Therefore, the sequential state discrimination problem is equivalent to the following optimization problem.

4 $$\begin{array}{ll}{\rm{maximize}} & {P}_{s}^{(B,C)}\\ {\rm{subjectto}} & ({\alpha }_{1},{\alpha }_{2},\cdots ,{\alpha }_{N})\,\in \,{\rm{int}}\,(C),\\  & ({\alpha ^{\prime} }_{1},{\alpha ^{\prime} }_{2},\cdots ,{\alpha ^{\prime} }_{N})\,\in \,\partial C^{\prime} .\end{array}$$

∂*C*′ relies on (*α*_1_, *α*_2_, …, *α*_*N*_). Now, we prove that Conjecture 1 and Conjecture 2 hold in cases of *N* = 2 and *N* = 3. And we investigate the optimization problems of Eq. (4) when *N* = 2 and *N* = 3. We expect that our approach can be extended to a case where *N* > 3.

### Examples: sequential state discrimination for two or three pure states

Suppose that Alice prepares a quantum state out of two(or three) pure quantum states^6–9,31–33^.

#### Two pure states(Qubits)

Assume that with a prior probability *q*_*i*_, Alice prepares a qubit state out of two pure qubit states |*ψ*_*i*_〉 ∈ {|*ψ*_1_〉,|*ψ*_2_〉} and sends it to Bob. The overlap between two qubit states is 〈*ψ*_1_|*ψ*_2_〉 = *s*exp (*iϕ*), *s* ≥ 0. When *s* ∈ [0, 1), there exists the inverse of the Gram matrix *G* for Alice’s quantum states. This implies the existence of Bob’s POVM discriminating Alice’s qubit state without error. Bob’s POVM can be represented by a two-dimensional real vector (*α*_1_, *α*_2_). (*α*_1_, *α*_2_) ∈ *C*, satisfying the conditions of Bob’s POVM (B1), (B2), (B3), fulfills the following inequality:

5 $$(1-{\alpha }_{1})(1-{\alpha }_{2})-{s}^{2}\ge 0.$$

The inequality is obtained by the non-negativity condition of $\tilde{G}={\{\langle {\psi }_{i}|{M}_{0}|{\psi }_{j}\rangle \}}_{i,j=1}^{2}$. Eq. (5) indicates that *C* is a convex set. One can show that the set *C* of (*α*_1_, *α*_2_) fulfilling Eq. (5) is convex, in the following way. The strict equality *α*_2_ = *f*(*α*_1_)(*f*(*x*) ≡ 1 + *s*^2^(*x* − 1)^−1^) of Eq. (5) is monotonic decreasing as *α*_1_ varies from 0 to 1−*s*^2^. In addition, *f*″(*α*_1_) of strict equality is negative-definite. Therefore, *C* surrounded by strict equality of Eq. (5) and *α*_1_ = 0, *α*_2_ = 0 is convex. Figure 1(a) displays the structure of C when the overlap *s* between two pure qubits, prepared by Alice is 0.3, which shows that *C* is convex. Because of the fact that in sequential state discrimination the post-measurement states of Bob should be considered, we need to find the Kraus operator *K*_*i*_ that corresponds to *M*_*i*_. When *i* = 1,2, from singular value decomposition^34^, we have ${K}_{i}=\sqrt{{\alpha }_{i}}|{\psi ^{\prime} }_{i}\rangle \langle {\tilde{\psi }}_{i}|$, where $|{\tilde{\psi }}_{i}\rangle ={\sum }_{j=1}^{2}{G}_{ji}^{-1}|{\psi }_{j}\rangle$ and $|{\psi ^{\prime} }_{i}\rangle$ is one of Bob’s post-measurement state. (It is not a general form of the Kraus operator^35^. However, because Charlie performs unambiguous discrimination, it is sufficient to consider a special type of Kraus operator.). Now, let us find the Kraus operator *K*_0_ corresponding to *M*_0_. *K*_0_ can be thought of as a linear map that sends two of Alice’s qubit states to linear independent states $|{\psi ^{\prime} }_{1}\rangle \,{\rm{and}}\,{\psi ^{\prime} }_{2}\rangle$,

6 $${K}_{0}=\sqrt{{\gamma }_{1}}|{\psi ^{\prime} }_{1}\rangle \langle {\tilde{\psi }}_{1}|+\sqrt{{\gamma }_{2}}|{\psi ^{\prime} }_{2}\rangle \langle {\tilde{\psi }}_{2}|.$$

where *γ*_1_, *γ*_2_ ≥ 0. The condition of ${M}_{0}={K}_{0}^{\dagger }{K}_{0}$ can be understood in terms of $\langle {\psi }_{i}|{M}_{0}\rangle |{\psi }_{j}\rangle =\langle {\psi }_{i}|{K}_{0}^{\dagger }{K}_{0}|{\psi }_{j}\rangle (\,\forall \,i,j)$, which gives $s^{\prime} =s/\sqrt{(1-{\alpha }_{1})(1-{\alpha }_{2})}(s^{\prime} =|\langle {\psi ^{\prime} }_{1}|{\psi ^{\prime} }_{2}\rangle |\rangle$. When Bob performs an optimal unambiguous discrimination, *s*′ becomes one(*s*′ = 1). This means that Charlie cannot discriminate Bob’s post-measurement states. Therefore, Bob should use a nonoptimal unambiguous discrimination. Then, the Gram matrix *G*′ of Bob’s post-measurement states has its inverse. Charlie’s POVM, which can discriminate Bob’s post-measurement states without error, corresponds to a two-dimensional real vector $({\alpha ^{\prime} }_{1},{\alpha ^{\prime} }_{2})$. The condition for $({\alpha ^{\prime} }_{1},{\alpha ^{\prime} }_{2})\,\in \,C^{\prime}$ is given by

7 $$(1-{\alpha ^{\prime} }_{1})(1-{\alpha ^{\prime} }_{2})-{s^{\prime} }^{2}\ge 0.$$

This tells us that *C*′ is a convex set and depends on *C*. In Eq. (7), *s*′ is a function of (*α*_1_, *α*_2_), being not a function of $({\alpha ^{\prime} }_{1},{\alpha ^{\prime} }_{2})$. One can show that the set *C*′ of $({\alpha ^{\prime} }_{1},{\alpha ^{\prime} }_{2})$ satisfying Eq. (7) is convex, in similar fashion of *C*. The fact that Bob can obtain an information from Alice’s quantum state implies that the success probability *α*_*i*_ is non-zero. From the relation between *s*′ and *s* for non-zero *α*_*i*_, we find a strict inequality *s*′ > *s*. As *s* increases, *f*(*x*) decreases and the size of *C*′ is smaller than that of *C*. From Fig. 1(b), one can see that *C*′ is convex and the size of *C*′ is smaller than that of *C*. Here, solid line(dashed line) displays ∂*C*′ when (*α*_1_, *α*_2_) = (0.5, 0.5)((*α*_1_, *α*_2_) = (0.5, 0.7)). As (*α*_1_, *α*_2_) approaches to ∂*C*, the size of *C*′ decreases, implying the trade-off phenomena which tells that more information Bob obtains from Alice’s pure states, less information Charlie gets. Therefore, we show that Conjecture 1 and 2 hold. Eqs (5) and (7) are the constraints of optimization problem (4). Because Bob(Charlie) uses a nonoptimal(optimal) unambiguous discrimination, Eqs (5) and (7) becomes a strict inequality(strict equality). If Bob does not optimally discriminate Alice’s pure qubit, (*α*_1_, *α*_2_) exists in the interior of *C*. Therefore, (*α*_1_, *α*_2_) satisfies the strict inequality of Eq. (5)^27^. In the similar way, if Charlie optimally discriminate the post-measurement state of Bob, $({\alpha ^{\prime} }_{1},{\alpha ^{\prime} }_{2})$ exists on ∂*C*′. Therefore, $({\alpha ^{\prime} }_{1},{\alpha ^{\prime} }_{2})$ satisfies the strict equality of Eq. (7)^27^. Then, Eq. (4) becomes the following optimization problem;

8 $$\begin{array}{ll}{\rm{maximize}} & {P}_{s}^{(B,C)}={q}_{1}{\alpha }_{1}{\alpha ^{\prime} }_{1}+{q}_{2}{\alpha }_{2}{\alpha ^{\prime} }_{2}\\ {\rm{subject}}\,{\rm{to}} & (1-{\alpha }_{1})(1-{\alpha }_{2})-{s}^{2} > 0,\\  & (1-{\alpha ^{\prime} }_{1})(1-{\alpha ^{\prime} }_{2})-{s^{\prime} }^{2}=0.\end{array}$$

If $({\alpha ^{\prime} }_{1},{\alpha ^{\prime} }_{2})$ are ${\alpha ^{\prime} }_{1},{\alpha ^{\prime} }_{2} > 0$, the best strategy for Charlie is to discriminate two of Bob’s post-measurement states. This implies that the best strategy for Bob and Charlie is to discriminate every of the two pure quantum state. In this case, the sequential discrimination scenario for Bob and Charlie is summarized as Theorem 1.

##### **Theorem 1**.

* Suppose that with a prior probability q*_*i*_*, Alice prepares a pure state* |*ψ*_*i*_〉 out of {|*ψ*_1_〉, *|ψ*_2_〉} *satisfying 〈ψ*_1_|*ψ*_2_〉 = *s exp*(*iϕ*), *s* ≥ 0. *The sequential state discrimination problem in which Bob and Charlie discriminate two pure states is equivalent to the following optimization problem*:

9 $$\begin{array}{ll}{\rm{maximize}}\, & {P}_{s}^{(B,C)}={q}_{1}{\alpha }_{1}+{q}_{2}{\alpha }_{2}-2s\sqrt{\frac{{q}_{1}{q}_{2}{\alpha }_{1}{\alpha }_{2}}{\mathrm{(1}-{\alpha }_{1}\mathrm{)(1}-{\alpha }_{2})}}\\ {\rm{subject}}\,{\rm{to}}\, & {\alpha }_{2} < \frac{{\alpha }_{1}\mathrm{(1}-{\alpha }_{1})}{x+{\alpha }_{1}\mathrm{(1}-{\alpha }_{1})},\,{\alpha }_{1} < \frac{{\alpha }_{2}\mathrm{(1}-{\alpha }_{2})}{y+{\alpha }_{2}\mathrm{(1}-{\alpha }_{2})},\,x={s}^{2}\frac{{q}_{2}}{{q}_{1}},\,y={s}^{2}\frac{{q}_{1}}{{q}_{2}}.\end{array}$$

The proof of Theorem. 1 is shown in the Methods.

Equation (9) is a nonlinear optimization problem and is a difficult to solve analytically. In special cases, the optimization problem can be solved analytically. Because the object function is continuous, when (*α*_1_, *α*_2_) is an optimization point, the gradient of the object function becomes zero. Therefore, the following equations hold at the optimization point:

10 $$\begin{array}{rcl}{\alpha }_{2} & = & \frac{{\alpha }_{1}{(1-{\alpha }_{1})}^{3}}{x+{\alpha }_{1}{(1-{\alpha }_{1})}^{3}},\,x={s}^{2}\frac{{q}_{2}}{{q}_{1}},\\ {\alpha }_{1} & = & \frac{{\alpha }_{2}{(1-{\alpha }_{2})}^{3}}{y+{\alpha }_{2}{(1-{\alpha }_{2})}^{3}},\,y={s}^{2}\frac{{q}_{1}}{{q}_{2}}.\end{array}$$

The inverse does not generally hold. However, when two prior probabilities are equal, two equations have an inverse function relationship. Then, the optimization condition becomes ${\alpha }_{1}={\alpha }_{2}=1-\sqrt{s}$, and the optimized success probability is ${\rm{\max }}\,{P}_{s}^{(B,C)}={\mathrm{(1}-\sqrt{s})}^{2}$, which agrees with the result of ref.^21^. The solution of Eq. (9), $({\alpha }_{1},{\alpha }_{2})=(1-\sqrt{s},1-\sqrt{s})$ is located in the interior of *C*. And the condition of Charlie for providing optimal success probability, $({\alpha ^{\prime} }_{1},{\alpha ^{\prime} }_{2})=\mathrm{(1}-\sqrt{\mathrm{s,}}\,1-\sqrt{s})$ exists on ∂*C*′. The fact that optimal condition for sequential state discrimination is ${\alpha }_{i}={\alpha ^{\prime} }_{i}=1-\sqrt{s}$ implies that with the same probability, Bob and Charlie, without error, discriminate *i*-th pure qubit, with supports the result of ref.^21^.

If two prior probabilities are not equal, it is difficult to analytically prove the argument of ref.^21^. However, it can be shown numerically^36,37^. For example, in the case of *q*_1_ = 0.55, *q*_2_ = 0.45, and *s* = 0.12, one can numerically find (*α*_1_, *α*_2_) = (0.721987, 0.568364), which is the solution of Eq. (10). Then, by Eq. (26) of Method, one can obtain $({\alpha ^{\prime} }_{1},{\alpha ^{\prime} }_{2})$, which is the same as (*α*_1_, *α*_2_). It should be noted that $({\alpha ^{\prime} }_{1},{\alpha ^{\prime} }_{2})$ = (0.721987, 0.568364) is located on ∂*C*′. In fact, the reason that the success probability of Bob and Charlie is the same is that even though Bob performs a nonoptimal unambigous discrimimination, Charlie should optimally discriminate Bob’s post measurement state.

Let us assume that ${\alpha ^{\prime} }_{2}=0$. Then, Charlie discriminates only $|{\psi ^{\prime} }_{1}\rangle$| without error. In this case, ${\alpha ^{\prime} }_{1}=1-{s^{\prime} }^{2}$ is obtained. Likewise, Bob also discriminates only *ψ*_1_, which is the best strategy (*α*_2_ = 0) for Bob and Charlie. Therefore, the problem of sequential state discrimination is equivalent to the following optimization problem:

11 $${\rm{maximize}}\,{P}_{s}^{(B,C)}={q}_{1}{\alpha }_{1}\{1-\frac{{s}^{2}}{1-{\alpha }_{1}}\}$$

The optimized condition is *α*_1_ = 1 − *s*. Then, one has ${\rm{\max }}\,{P}_{s}^{(B,C)}={q}_{1}{(1-s)}^{2}$. When Bob and Charlie discriminate only |*ψ*_2_〉(i.e., *α*_1_ = ${\alpha ^{\prime} }_{1}=0$), the optimized condition becomes *α*_2_ = 1 − *s*. Additionally, one has ${\rm{m}}{\rm{a}}{\rm{x}}{P}_{s}^{(B,C)}={q}_{2}{\mathrm{(1}-s)}^{2}$. Therefore, the optimized success probability that in sequential state discrimination Bob and Charlie discriminate only one of Alice’s two pure qubit states, becomes max{*q*_1_(1 − *s*^2^), *q*_2_(1 − *s*^2^)}. The case of *q*_1_ = *q*_2_ contains the result of Pang *et al*.^23^.

If general prior probabilities ${q}_{1}\ne {q}_{2}$ are considered, one must numerically find (*α*_1_, *α*_2_) satisfying Eq. (10). In this report, a random search method^36^ based on Monte Carlo methods is used. By these methods, one can search almost entire (*α*_1_, *α*_2_), fulfilling the constraint of Eq. (9).

When Bob can obtain a partial information on Alice’s qubit, since the overlap between Bob’s post measurement states should be increased, *s*′ is always greater than *s*. Therefore, the size of convex set *C*′ is smaller than that of *C*. When Bob performs an optimal unambiguous discrimination, because of *s*′ = 1, the element of *C*′ is only a zero vector. This implies that Charlie cannot obtain any information from Bob’s post-measurement state.

#### Three pure states(Qutrits)

Now let us consider the case of three pure qutrit states. With a prior probability *q*_*i*_, Alice prepares an element |*ψ*_*i*_〉 out of a set of qutrits {|*ψ*_1_〉, |*ψ*_2_〉, |*ψ*_3_〉} and sends it to Bob. The overlap between those qutrit states is given by 〈*ψ*_*i*_|*ψ*_*j*_〉 = *s*_*k*_exp(*iε*_*ijk*_*ϕ*_*k*_), *s*_*k*_ ≥ 0, where *ε*_*ijk*_ is the Levi-Civita symbol. Let the sub-matrices of $\tilde{G}={\{\langle {\psi }_{i}|{M}_{0}|{\psi }_{j}\rangle \}}_{i.j=1}^{3}$ be ${\tilde{G}}_{1},{\tilde{G}}_{2},{\tilde{G}}_{3}$. When ${\rm{\det }}\,\tilde{G}\ge 0,{\tilde{G}}_{k}\ge 0,\langle {\psi }_{k}|{M}_{0}|{\psi }_{k}\rangle \ge 0$, $\tilde{G}$ is positive-semidefinite^32,33^. This condition indicates that three dimensional real vector (*α*_1_, *α*_2_, *α*_3_) ∈ *C* corresponding to Bob’s POVM satisfies the following relationship:

12 $$\begin{array}{c}{\bar{\alpha }}_{1}{\bar{\alpha }}_{2}{\bar{\alpha }}_{3}-{s}_{1}^{2}{\bar{\alpha }}_{1}-{s}_{2}^{2}{\bar{\alpha }}_{2}-{s}_{3}^{2}{\bar{\alpha }}_{3}+2{s}_{1}{s}_{2}{s}_{3}\,\cos \,{\rm{\Phi }}\ge 0,\\ {\bar{\alpha }}_{i}{\bar{\alpha }}_{j}-{s}_{k}^{2}\ge 0.\,\forall i\ne j\ne k.\end{array}$$

Here, ${\bar{\alpha }}_{i}=1-{\alpha }_{i}$ and Φ = *ϕ*_1_ + *ϕ*_2_ + *ϕ*_3_ are refered to as a geometric phase^32^. The set of (*α*_1_, *α*_2_, *α*_3_) satisfying Eq. (12) is convex. Let us obtain Kraus operator *K*_*i*_, corresponding to Bob’s POVM. If *i* = 1, 2, 3, the necessary and sufficient condition for ${M}_{i}={K}_{i}^{\dagger }{K}_{i}$ is ${K}_{i}={\alpha }_{i}|{\psi ^{\prime} }_{i}\rangle \langle {\tilde{\psi }}_{i}|$, where $|{\tilde{\psi }}_{i}\rangle ={\sum }_{j=1}^{3}{G}_{ji}^{-1}|{\psi }_{j}\rangle$ and $|{\psi ^{\prime} }_{i}\rangle$ is a Bob’s post-measurement state. For *i* = 0, let us assume the Kraus operator as follows:

13 $${K}_{0}=\sqrt{{\gamma }_{1}}|{\psi ^{\prime} }_{1}\rangle \langle {\tilde{\psi }}_{1}|+\sqrt{{\gamma }_{2}}|{\psi ^{\prime} }_{2}\rangle \langle {\tilde{\psi }}_{2}|+\sqrt{{\gamma }_{3}}|{\psi ^{\prime} }_{3}\rangle \langle {\tilde{\psi }}_{3}|.$$

Because *K*_0_ is a linear map sending |*ψ*_*i*_〉 to |*ψ*_*i*′_〉, from the condition of ${M}_{0}={K}_{0}^{\dagger }{K}_{0}$ and$\langle {\psi }_{i}|{M}_{0}|{\psi }_{j}\rangle =\langle {\psi }_{i}|{K}_{0}^{\dagger }{K}_{0}$ $|{\psi }_{j}\rangle (\,\forall \,i,j)$, $\langle {\psi ^{\prime} }_{i}|{\psi ^{\prime} }_{j}\rangle =\langle {\psi }_{i}|{\psi }_{j}\rangle /\sqrt{(1-{\alpha }_{i})(1-{\alpha }_{j})}$ is obtained. Assuming that $\langle {\psi ^{\prime} }_{i}|{\psi ^{\prime} }_{j}\rangle ={s^{\prime} }_{k}\,\exp \,(i{\varepsilon }_{ijk}{\varphi ^{\prime} }_{k})$, one has the relations of ${s^{\prime} }_{k}={s}_{k}/\sqrt{(1-{\alpha }_{i})(1-{\alpha }_{j})}$, ${\varphi ^{\prime} }_{k}={\varphi }_{k}(\,\forall \,i\ne j\ne k)$. When (*α*_1_, *α*_2_, *α*_3_) exists in the interior of *C*, the Gram matrix *G*′, composed of the post-measurement states of Bob, has an inverse matrix. Therefore, there exists Charlie’s POVM, discriminating the post-measurement states of Bob without error. Like Bob’s POVM, the conditions of Charlie’s POVM are given by

14 $$\begin{array}{c}{\bar{\alpha }^{\prime} }_{1}{\bar{\alpha }^{\prime} }_{2}{\bar{\alpha }^{\prime} }_{3}-{s^{\prime} }_{1}^{2}{\bar{\alpha }^{\prime} }_{1}-{s^{\prime} }_{2}^{2}{\bar{\alpha }^{\prime} }_{2}-{s^{\prime} }_{3}^{2}{\bar{\alpha }^{\prime} }_{3}+2{s^{\prime} }_{1}{s^{\prime} }_{2}{s^{\prime} }_{3}\,\cos \,{\rm{\Phi }}\ge 0,\\ {\bar{\alpha }^{\prime} }_{i}{\bar{\alpha }^{\prime} }_{j}-{s^{\prime} }_{k}^{2}\ge 0.\,\forall \,i\ne j\ne k.\end{array}$$

where ${\bar{\alpha }^{\prime} }_{i}=1-{\alpha ^{\prime} }_{i}$. The set *C*′ of three-dimensional real vectors satisfying Eq. (14) is convex and depends on Bob’s POVM. Therefore, Conjecture 1 and 2 hold in the sequential state discrimination of the three qutrit states. Because Charlie performs an optimal unambiguous discrimination, $({\alpha ^{\prime} }_{1},{\alpha ^{\prime} }_{2},{\alpha ^{\prime} }_{3})$ exists on the surface of Eq. (14). The sequential state discrimination problem for three qutrit states by Bob and Charlie becomes the following optimization problem:

15 $$\begin{array}{ll}{\rm{maximize}} & {P}_{s}^{(B,C)}={q}_{1}{\alpha }_{1}{\alpha ^{\prime} }_{1}+{q}_{2}{\alpha }_{2}{\alpha ^{\prime} }_{2}+{q}_{3}{\alpha }_{3}{\alpha ^{\prime} }_{3}\\ {\rm{subjectto}} & ({\alpha }_{1},{\alpha }_{2},{\alpha }_{3})\in \,{\rm{int}}\,(C),\\  & ({\alpha ^{\prime} }_{1},{\alpha ^{\prime} }_{2},{\alpha ^{\prime} }_{3})\in \partial C^{\prime} .\end{array}$$

Here, int (*C*) is a set of (*α*_1_, *α*_2_, *α*_3_) strictly satisfying Eq. (12). ∂*C*′ is a set of (*α*_1_, *α*_2_, *α*_3_) fulfilling ${\rm{\det }}\,\tilde{G}=0$ in Eq. (14). The sequential state discrimination of three qutrit states for Bob and Charlie can be categorized by the number of discriminations of qutrit states by Bob and Charlie. (The theorems are proven in the Methods).

##### **Theorem 2**.

* Alice prepares a element* |*ψ*_*i*_〉*, with a prior probability q*_*i*_*, from three pure qutrit states* {|*ψ*_1_〉, |*ψ*_2_〉, |*ψ*_3_〉} *satisfying* 〈*ψ*_*i*_|*ψ*_*j*_〉 = *s*_*k*_ exp (*iε*_*ijk*_*ϕ*_*k*_), *s*_*k*_ ≥ 0 *and sends it to Bob. If in sequential state discrimination, Bob and Charlie discriminate only a single qutrit state* (*for example*, $${\alpha }_{2}={\alpha }_{3}=0,{\alpha ^{\prime} }_{2}={\alpha ^{\prime} }_{3}=0$$) *out of three qutrit states, the optimal conditions for the sequential state discrimination are* $${\alpha }_{i}=1-\sqrt{{\chi }_{i}({\rm{\Phi }})}$$ *and* $${\alpha ^{\prime} }_{i}=1-{\chi }_{i}({\rm{\Phi }})$$, *where* $${\chi }_{i}({\rm{\Phi }})=({s}_{j}^{2}+{s}_{k}^{2}-2\,{s}_{i}{s}_{j}{s}_{k}\,\cos \,{\rm{\Phi }})/(1-{s}_{i}^{2})$$. *Then, the optimized success probability becomes* $${\rm{\max }}\,{P}_{s}^{(B,C)}={{\rm{\max }}}_{i}\{{q}_{i}$$$$\mathrm{(1}-{\chi }_{i}({\rm{\Phi }}))\}$$.

##### **Theorem 3**.

* Suppose that Alice prepares a element* |*ψ*_*i*_〉*, with a prior probability q*_*i*_*, from three pure qutrit states* {|*ψ*_1_〉, |*ψ*_2_〉, |*ψ*_3_〉} *satisfying* 〈*ψ*_i_|*ψ*_j_〉* = s*_*k*_ exp (*iε*_*ijk*_*ϕ*_*k*_), *s*_*k*_ ≥ 0 *and sends it to Bob. If in sequential state discrimination, Bob and Charlie discriminate only two qutrit states (for example*, ${\alpha }_{3}=0,{\alpha ^{\prime} }_{3}=0$*) out of three qutrit states, the solution for the sequential state discrimination is equivalent to that of the following optimization problem*:

16 $$\begin{array}{ll}{\rm{maximize}} & {P}_{s}^{(B,C)}={q}_{i}{\alpha }_{i}(1-\frac{{s}_{j}^{2}}{1-{\alpha }_{i}})+{q}_{j}{\alpha }_{j}(1-\frac{{s}_{i}^{2}}{1-{\alpha }_{j}})-2\sqrt{\frac{{q}_{i}{q}_{j}{\alpha }_{i}{\alpha }_{j}}{\mathrm{(1}-{\alpha }_{i}\mathrm{)(1}-{\alpha }_{j})}}{\xi }_{ij}({\rm{\Phi }})\\ {\rm{subject}}\,{\rm{to}} & {\alpha }_{j} < \frac{{\alpha }_{i}{\mathrm{(1}-{\alpha }_{i}-{s}_{j}^{2})}^{2}}{{\alpha }_{i}{\mathrm{(1}-{\alpha }_{i}-{s}_{j}^{2})}^{2}+({q}_{j}/{q}_{i}){\xi }_{ij}^{2}({\rm{\Phi }}\mathrm{)(1}-{\alpha }_{i})},\\  & {\alpha }_{i} < \frac{{\alpha }_{j}{\mathrm{(1}-{\alpha }_{j}-{s}_{i}^{2})}^{2}}{{\alpha }_{j}{\mathrm{(1}-{\alpha }_{j}-{s}_{i}^{2})}^{2}+({q}_{i}/{q}_{j}){\xi }_{ij}^{2}({\rm{\Phi }}\mathrm{)(1}-{\alpha }_{j})}.\end{array}$$

where ${\xi }_{ij}({\rm{\Phi }})=\sqrt{{s}_{i}^{2}{s}_{j}^{2}+{s}_{k}^{2}-2{s}_{i}{s}_{j}{s}_{k}\,\cos \,{\rm{\Phi }}}$ and *every index satisfies* $i\ne j\ne k$,

When (*α*_1_, *α*_2_) is an optimal pint of Eq. (16), the gradient of the object function at the point is zero. Therefore, the optimal condition is given by

17 $$\begin{array}{rcl}{\alpha }_{j} & = & \frac{{\alpha }_{i}{\{{s}_{j}^{2}-{(1-{\alpha }_{i})}^{2}\}}^{2}}{{\alpha }_{i}{\{{s}_{j}^{2}-{(1-{\alpha }_{i})}^{2}\}}^{2}+({q}_{j}/{q}_{i}){\xi }_{ij}^{2}({\rm{\Phi }})(1-{\alpha }_{i})},\\ {\alpha }_{i} & = & \frac{{\alpha }_{j}{\{{s}_{i}^{2}-{(1-{\alpha }_{j})}^{2}\}}^{2}}{{\alpha }_{j}{\{{s}_{i}^{2}-{(1-{\alpha }_{j})}^{2}\}}^{2}+({q}_{i}/{q}_{j}){\xi }_{ij}^{2}({\rm{\Phi }})(1-{\alpha }_{j})},\,\forall \,i\ne j\ne k.\end{array}$$

However, the inverse does not hold. When *q*_*i*_ = *q*_*j*_ and *s*_1_ = *s*_2_ = *s*_3_ = *s*, the equations have an inverse function relationship. Then, the optimal condition is obtained by ${\alpha }_{i}={\alpha }_{j}=1-\sqrt{{s}^{2}+\xi ({\rm{\Phi }})}$ (∀*ξ*(Φ) = *ξ*_*ij*_(Φ)). If (*α*_*i*_, *α*_*j*_) does not fulfill Eq. (16), the best strategy for Bob and Charlie is to discriminate only a single qutrit state out of three pure qutrit states.

##### **Theorem 4**.

* Suppose that Alice prepares an element* |*ψ*_*i*_〉*, with a prior probability q*_*i*_*, from three pure qutrit states* {|*ψ*_1_〉, |*ψ*_2_〉, |*ψ*_3_〉} *satisfying* 〈*ψ*_*i*_|*ψ*_j_〉 = *s*_*k*_ exp (*iε*_*ijk*_*ϕ*_*k*_), *s*_*k*_ ≥ 0 *and sends it to Bob. When* Φ = *ϕ*_1_ + *ϕ*_2_ + *ϕ*_3_ = 0 *is fulfilled, if Bob and Charlie discriminate every three qutrit state in sequential state discrimination, the solution for the sequential state discrimination is equivalent to that of the following optimization problem*:

18 $$\begin{array}{ll}{\rm{maximize}} & {P}_{s}^{(B,C)}={q}_{1}{\alpha }_{1}+{q}_{2}{\alpha }_{2}+{q}_{3}{\alpha }_{3}-2{s}_{1}\sqrt{\frac{{q}_{2}{q}_{3}{\alpha }_{2}{\alpha }_{3}}{\mathrm{(1}-{\alpha }_{2}\mathrm{)(1}-{\alpha }_{3})}}\\  & -\,2{s}_{2}\sqrt{\frac{{q}_{1}{q}_{3}{\alpha }_{1}{\alpha }_{3}}{\mathrm{(1}-{\alpha }_{1}\mathrm{)(1}-{\alpha }_{3})}}+2{s}_{3}\sqrt{\frac{{q}_{1}{q}_{2}{\alpha }_{1}{\alpha }_{2}}{\mathrm{(1}-{\alpha }_{1}\mathrm{)(1}-{\alpha }_{2})}}\\ {\rm{subject}}\,{\rm{to}} & ({\alpha }_{1},{\alpha }_{2},{\alpha }_{3})\,\in \,{\rm{int}}(C),({\alpha ^{\prime} }_{1},{\alpha ^{\prime} }_{2},{\alpha ^{\prime} }_{3})\,\in \,\partial C^{\prime} .\end{array}$$

In addition, from the solution discussed in ref.^32^, we can find the optimal condition for Charlie, which is ${\alpha ^{\prime} }_{i}=1-({s}_{j}{s}_{k}/{s}_{i}){(1-{\alpha }_{i})}^{-1}(i\ne j\ne k)$. The sequential state discrimination of the case becomes the following optimization problem.

19 $${\rm{maximize}}\,{P}_{s}^{(B,C)}={q}_{1}{\alpha }_{1}\{1-\frac{{s}_{2}{s}_{3}}{{s}_{1}}\frac{1}{1-{\alpha }_{1}}\}+{q}_{2}{\alpha }_{2}\{1-\frac{{s}_{1}{s}_{3}}{{s}_{2}}\frac{1}{1-{\alpha }_{2}}\}+{q}_{3}{\alpha }_{3}\{1-\frac{{s}_{1}{s}_{2}}{{s}_{3}}\frac{1}{1-{\alpha }_{3}}\}$$

The optimal solution of Eq. (19) is ${\alpha }_{i}=1-\sqrt{{s}_{j}{s}_{k}/{s}_{i}}(i\ne j\ne k)$. The solution satisfies every equality of Eq. (14)^32^. When the overlap between every pure state is the same, the result in this report contains those of M. Hillery and J. Mimih^26^.

Also, the success probability of Charlie is ${\alpha ^{\prime} }_{i}={\alpha }_{i}=1-\sqrt{{s}_{j}{s}_{k}/{s}_{i}}$, which is the same as Bob’s. Here, $({\alpha }_{1},{\alpha }_{2},{\alpha }_{3})=$ $(1-\sqrt{{s}_{2}{s}_{3}/{s}_{1}},1-\sqrt{{s}_{1}{s}_{3}/{s}_{2}},1-\sqrt{{s}_{1}{s}_{2}/{s}_{3}})$ is located in the interior of *C* and $({\alpha ^{\prime} }_{1},{\alpha ^{\prime} }_{2},{\alpha ^{\prime} }_{3})=(1-\sqrt{{s}_{2}{s}_{3}/{s}_{1}},$ $1-\sqrt{{s}_{1}{s}_{3}/{s}_{2}},1-\sqrt{{s}_{1}{s}_{2}/{s}_{3}})$ exists on ∂*C*′.

However, for a arbitrary geometric phase Φ, the optimization problem cannot be analytically presented, as in the case of Theorem 4. A detailed explanation is given in the Methods.

Now, let us investigate the structure of *C* and *C*′. It can be shown that *C* and *C*′ are convex. The vertices of *C* and *C*′ are given by

20 $$\begin{array}{rcl}{\alpha }_{i,{\rm{\max }}} & = & 1-\frac{{s}_{j}^{2}+{s}_{k}^{2}-2{s}_{i}{s}_{j}{s}_{k}\,\cos \,{\rm{\Phi }}}{1-{s}_{i}^{2}},\\ {\alpha ^{\prime} }_{i,{\rm{\max }}} & = & 1-\frac{{s^{\prime} }_{j}^{2}+{s^{\prime} }_{k}^{2}-2{s^{\prime} }_{i}{s^{\prime} }_{j}{s^{\prime} }_{k}\,\cos \,{\rm{\Phi }}}{1-{s}_{i}^{^{\prime} 2}}\\  & = & 1-\,\frac{{s}_{j}^{2}\mathrm{(1}-{\alpha }_{j})+{s}_{k}^{2}\mathrm{(1}-{\alpha }_{k})-2{s}_{i}{s}_{j}{s}_{k}\,\cos \,{\rm{\Phi }}}{\mathrm{(1}-{\alpha }_{i}\mathrm{)\{(1}-{\alpha }_{j}\mathrm{)(1}-{\alpha }_{k})-{s}_{i}^{2}\}},\,\forall \,i\ne j\ne k.\end{array}$$

Three vertices of *C*′ depend on Bob’s POVM. According to Eq. (19), 0 ≤ *α*_*i*,max_ ≤ 1. *α*_*i*,max_ ≤ 1 can be easily proven. *α*_*i*,max_ ≥ 0 is equivalent to a positive-semidefiniteness condition of the Gram matrix. Similarly, ${\alpha ^{\prime} }_{i,{\rm{\max }}}\le 1$ can be shown. ${\alpha ^{\prime} }_{i,{\rm{\max }}}$ is a decreasing function to (*α*_1_, *α*_2_, *α*_3_). When *α*_1_ = *α*_2_ = *α*_3_ = 0, ${\alpha }_{i,{\rm{\max }}}={\alpha ^{\prime} }_{i,{\rm{\max }}}$ holds. Therefore, if (*α*_1_, *α*_2_, *α*_3_) satisfies the condition of Bob’s POVM, one can find ((*α*_1_, *α*_2_, *α*_3_) ∈ *C* → ∀*α*_*i*_ < 1) ${\alpha ^{\prime} }_{\mathrm{1,}{\rm{\max }}} < {\alpha }_{\mathrm{1,}{\rm{\max }}}$. This implies that the size of the convex set *C*′ for Charlie is smaller than that of *C*. When Bob performs an optimal unambiguous discrimination, one has ${\alpha ^{\prime} }_{i,{\rm{\max }}}=0$, which means that Charlie cannot obtain any information from Bob’s post-measurement state. In Fig. 2, Fig. 2(a) displays the Bob’s convex set *C* when *s*_1_ = 0.2, *s*_2_ = 0.3, *s*_3_ = 0.25. In Fig. 2(b), solid line(dashed line) indicates the boundary of Charlie’s convex set *C*′ when *α*_1_ = *α*_2_ = *α*_3_ = 0.5(*α*_1_ = *α*_2_ = 0.5, *α*_3_ = 0.7). Figure 2 clearly shows that *C* and *C*′ are convex. Furthermore, Fig. 2(b) shows that as (*α*_1_, *α*_2_, *α*_3_) approaches to its boundary ∂*C*, the size of Charlie’s convex set becomes smaller.

**Figure 3:**
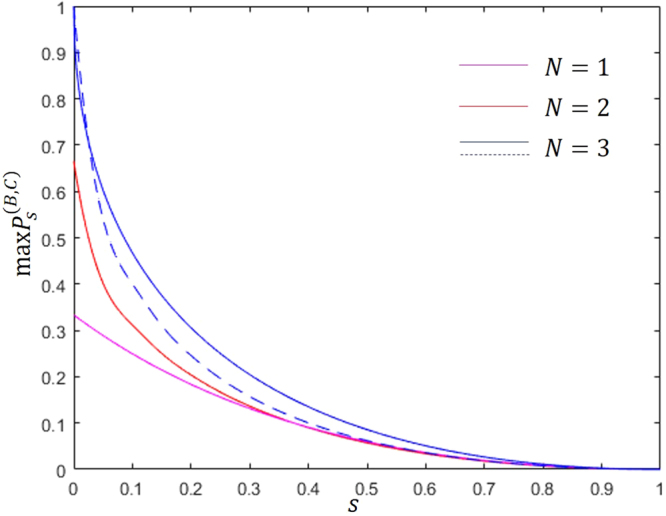
Optimal success probability of the sequential state discrimination of three linearly independent symmetric qutrits in terms of overlap *s*. Here, the prior probability of the three qutrits is assumed to be equal, and *N* denotes the number of pure qutrit states which Bob and Charlie discriminate. The blue solid line(blue dashed line) displays the optimal success probability of Eqs (18 and 19).

It is difficult to analytically solve the optimization problem described by Theorem 4. Unlike the case for two qubits, it is difficult to find an analytic solution for the sequential state discrimination of three qutrit states even in a symmetric case. Therefore, we use a random search method^36^ to obtain a numerical solution. Figure 3 displays the optimal success probability when *q*_1_ = *q*_2_ = *q*_3_ and *s*_1_ = *s*_2_ = *s*_3_ = *s*. Figure 3 shows that it is better for Bob and Charlie to discriminate every three pure qutrit state than it is for them to discriminate one(or two) pure qutrit state(s). This is different from the case involving two pure qubit states. In the case of two pure qubit states prepared with equal prior probability, when the overlap between the two qubit states is greater than $3-2\sqrt{2}$, discriminating only single qubit state is the best method^21,23^. However, for quantum key distribution, receivers should discriminate all quantum states prepared by a sender^21,38^. Therefore, our results for three pure qutrit states demonstrates the advantages of sequential state discrimination in quantum key distribution.

In addition, Hillery and Mimih^26^ studied the specific cases of sequential state discrimination. Even though we do not explain in detail, some examples of M. Hillery and J. Mimih^26^ can be understood by application of results of this report.

### Comparison with an other scenario

Here, we compare the sequential state discrimination of three pure qutrit states with quantum probabilistic cloning strategy^39^. Unlike sequential state discrimination, the method allows a classical communication.

- (*Quantum probabilistic cloning)* Suppose that Alice prepares a quantum state |*ψ*_*i*_〉(*i* ∈ {1, …, *N*}) with a prior probability *q*_*i*_ and sends it to Bob. Bob clones this quantum state probabilistically^39^. When Bob successfully copies the state, the quantum state after Bob’s copying becomes |*ψ*_*i*_〉 ⊗|*ψ*_*i*_〉. Bob shares |*ψ*_*i*_〉 ⊗ |*ψ*_*i*_〉 with Charlie. Bob and Charlie perform an optimal unambiguous discrimination on his own state, respectively. If Bob fails to copy the quantum state of Alice, he tells Charlie that he fails to copy the quantum state of Alice.

Even though there is no unitary operation for copying non-orthogonal quantum states^40^, one can clone a quantum state imperfectly^41^. There are many methods for copying a quantum state^39,42,43^. L. M. Duan *et al*^39^. showed that non-orthogonal pure quantum state can be cloned through Quantum probabilistic cloning.

#### **Theorem 5**.

(L. M. Duan *et al*^39^.) *Suppose that for a element* |*ψ*_*i*_〉 *of a set composed of non-orthogonal pure states* {|*ψ*_1_〉, …, |*ψ*_*N*_〉}, *one can clone a quantum state* |*ψ*_*i*_〉 ⊗ |*R*〉 → |*ψ*_*i*_〉 ⊗ |*ψ*_*i*_〉 *with a probability γ*_*i*_. *The necessary and sufficient condition for such quantum operation is that* $X-\sqrt{{\rm{\Gamma }}}Y\sqrt{{\rm{\Gamma }}}$ *becomes positive-semidefinite, where* $X={\{{\langle {\psi }_{i}|\psi \rangle }_{j}\}}_{i,j=1}^{N}$, $Y={\{\langle {\psi }_{i}|{\psi }_{j}{\rangle }^{2}\}}_{i,j=1}^{N}$, and $\sqrt{{\rm{\Gamma }}}=diag\{{\sqrt{\gamma }}_{1},\cdots ,{\sqrt{\gamma }}_{N}\}$^39,44^.

Let us assume equal probability for successfully copying a quantum state (*γ*_*i*_ = *γ* ∀*i*). Then, the probability for successful quantum probabilistic cloning of Bob and Charlie is given by

21 $${P}_{s,clone}^{(B,C)}=\sum _{i=1}^{N}{\rm{\Pr }}[|{\psi }_{i}\rangle ]\times {\rm{\Pr }}[{\rm{clone}}|{\psi }_{i}\rangle ]\times {\rm{\Pr }}[i|{\rm{clone}}]$$

Here, *Pr*[|*ψ*_*i*_〉] is a prior probability with which Alice prepares |*ψ*_*i*_〉. Pr[clone|*ψ*_*i*_〉] is the probability that Bob successfully copies the quantum state |*ψ*_*i*_〉 of Alice. Pr[*i*|clone] is the probability that Bob and Charlie obtain measurement result *i*. The necessary and sufficient condition for Theorem 5 is that the pure states are linearly independent^39,44^. Therefore, there exists a POVM of Bob and Charlie that can discriminate Alice’s quantum states without error.

Suppose that Alice prepares a quantum state out of three linearly independent pure qutrits {|*ψ*_1_〉, |*ψ*_*2*_〉, |*ψ*_3_〉}. When Bob successfully copies the quantum state of Alice, Bob and Charlie independently discriminate Alice’s quantum state. Therefore, the best way to perform a strategy of probabilistic quantum cloning is for Bob and Charlie to perform an optimal unambiguous discrimination. Therefore, the real vector corresponding to an optimal POVM of Bob and Charlie is (*α*_1_, *α*_2_, *α*_3_). Then, the optimal success probability of Bob and Charlie, when probabilistic quantum cloning is imposed, becomes

22 $${P}_{s,clone}^{(B,C)}={\gamma }_{{\rm{opt}}}({q}_{1}{\alpha }_{1}^{2}+{q}_{2}{\alpha }_{2}^{2}+{q}_{3}{\alpha }_{3}^{2})$$

Here, *γ*_opt_ is a maximal *γ* satisfying Theorem 5. The optimal value of *γ* depends on the overlap between pure states prepared by Alice. The success probability of probabilistic quantum cloning is a convex function of (*α*_1_, *α*_2_, *α*_3_). Therefore, (*α*_1_, *α*_2_, *α*_3_) exists on the boundary ∂*C* of *C*.

#### **Example**.

Suppose that the overlap between three pure qutrits {|*ψ*_1_〉, |*ψ*_2_〉, |*ψ*_3_〉} can be given by

23 $$\langle {\psi }_{1}|{\psi }_{2}\rangle =-\,0.1,\,\langle {\psi }_{2}|{\psi }_{3}\rangle =0.1,\,\langle {\psi }_{3}|{\psi }_{1}\rangle =-\,s$$

**Figure 4:**
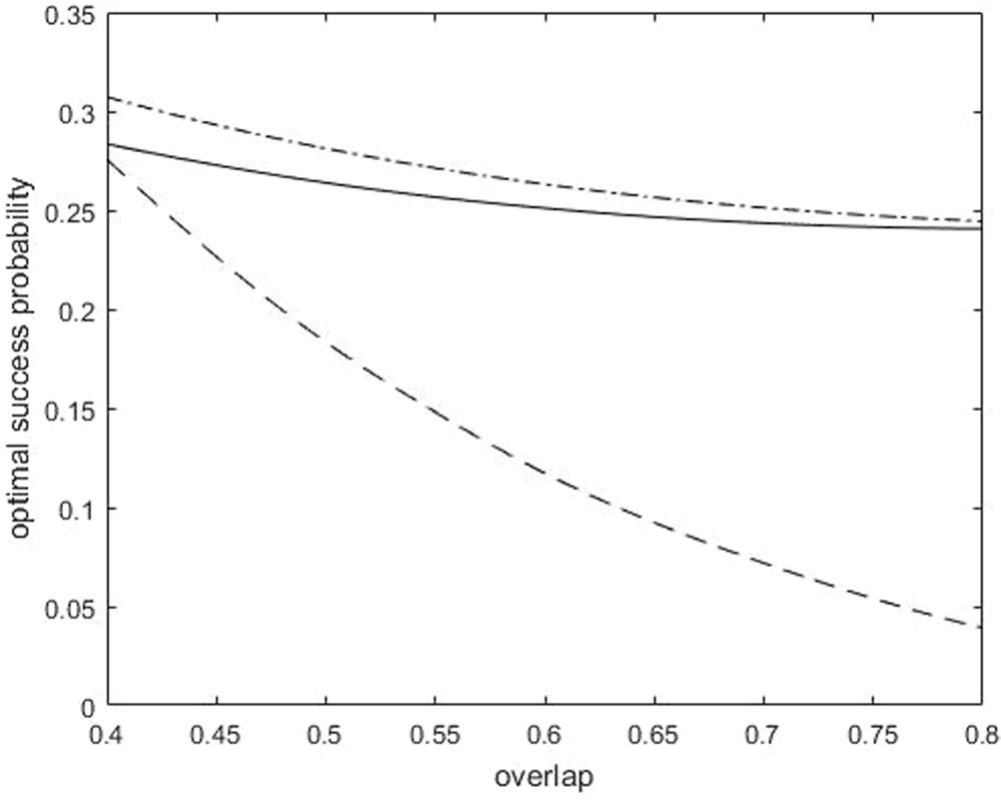
The optimal success probabilities of probabilistic quantum cloning and sequential state discrimination for three pure qutrit states. Here, the solid line(dash-dot line) shows the optimal success probability of sequential state discrimination based on Eqs (18 and 19), and the dashed line shows that of probabilistic quantum cloning.

Suppose that the prior probabilities for the three pure qutrits are *q*_1_ = *q*_3_ = 0.35, and *q*_2_ = 0.3. Let us assume that the geometric phase of the three pure qutrits is *ϕ*_1_ + *ϕ*_2_ + *ϕ*_3_ = 0 + *π* + *π* = 2*π*. Figure 4 displays the optimal success probabilities of probabilistic quantum cloning and sequential state discrimination, where the overlap *s* is *s* ∈ [0.4, 0.8]. According to Fig. 4, sequential state discrimination, unlike probabilistic quantum cloning, has a non-zero optimal success probability even when *s* becomes large. This is different from the case of two pure qubits with equal prior probabilities^21^. This implies that sequential state discrimination performs better than a probabilistic quantum cloning strategy.

### Revisiting The Security of Protocol Based on Sequential State Discrimination Scenario

Based on B92 protocol^38^, the security of B92 protocol can be obtained when Bob performs an unambiguous discrimination on nonorthogonal quantum states encoded by a sender Alice^21^. However, if an eavesdropper Eve exists between Alice and Bob, then the conclusive result of Bob will contain an error. When a discrepancy occurs from the comparison between Alice’s quantum state and Bob’s conclusive result, Alice and Bob can notice the existence of eavesdropper Eve.

Note that Alice and Bob are separated in space. Alice should inform Bob of the prior probability, in order for Bob to perform a unambiguous discrimination. In this process, if Alice uses a classical communication, Eve can obtain the prior probability without being noticed. When Eve optimally discriminates two quantum states of Alice, the post-measurement states of Eve are entirely overlapped, which implies that Bob cannot obtain any information from the post-measurement state of Eve. Therefore, Bob’s result is always inconclusive. Meanwhile, unless Bob notices Eve, Eve’s measurement should be identity. This implies that Eve cannot obtain any information from Alice’s quantum state. When Eve obtains a partial information from Alice’s quantum state, the overlap between Eve’s post-measurement states is larger than the overlap between Alice’s quantum states. Therefore, Bob’s conclusive result may contain an error with a probability.

In fact, this argument can be applied to the case of sequential state discrimination of three pure qutrit states. Now, we can have the following Conjecture.

#### **Conjecture 3**.

*In sequential discrimination strategy, when a receiver can obtain a partial information about a sender’s quantum state through a POVM, the size of convex set C*′ *of a second receiver is smaller than that of C of a first receiver*.

In fact, SSD strategy provides a protocol for distribution of secure key to multi-parties^21^, based on B92 protocol^38^. When an eavesdropper Eve tries to obtain a partial information between Alice and Bob, the conclusive result of Bob inevitably contains an error. In the similar way, if Eve gains an information between Bob and Charlie, the conclusive result of Charlie should have an error. It implies that whenever Eve obtains an information, Bob and Charlie can notice the existence of eavesdropper Eve. Therefore, Alice, Bob, and Charlie can share a secure key.

In addition, the example of our report shows that the proposed SSD strategy can provide a higher success probability than probabilistic quantum cloning strategy. We should note that in two qubit case, SSD strategy is not better than probabilistic quantum cloning strategy^21^. Therefore, when Alice encodes an information using non-orthogonal pure qutrits, SSD strategy can be more efficient one for QKD than probabilistic quantum cloning. It implies that SSD strategy can be a good candidate for multi-party QKD^27^.

Furthermore, if one uses larger number of linearly independent pure qudits, Alice can share more secure message with multi-parties^26^. Ref.^26^ considers only symmetric pure qudits. The result of our report may provide a strategy using general(nonsymmetric) pure qudits, for QKD of multi-parties.

## Discussion

In this report, we studied the sequential state discrimination of *N* pure states. One of advantages of the sequential state discrimination is that a sequential receiver Charlie can determine the pure state of a sender Alice, handling the post-measurement of a previous receiver Bob rather than the pure state of Alice. First, we provided a general formulation of sequential state discrimination for *N* pure states when Alice prepares a quantum state out of *N* pure states. Second, we obtained the condition for sequential state discrimination of two qubits with arbitrary prior probabilities. Third, we showed that when Alice prepares three qutrits with the identical prior probabilities, the best way for Bob and Charlie to perform sequential state discrimination of three qutrits is to unambiguously discriminate every three qutrit. This differs from the two qubits case, where the best sequential state discrimination method is not to discriminate every two qubit. In addition, we showed that the sequential state discrimination of three pure qutrit states performs better than probabilistic quantum cloning strategy.

If the three conjectures of our report can hold in the case of *N* qudits, we have a formulation of sequential state discrimination to *N* linearly independent qudits. In addition, we may generalize our sequential state discrimination approach to *N* parties. It is interesting to compare our strategy of *N* qudits for *N* parties with other strategies.

## Methods

In this section, we prove Theorem 1–4.

### **Proof of Theorem 1**.

When Alice prepares one of two pure qubits, the optimization problem of Eq. (4) is formulated by

24 $$\begin{array}{rcl}{\rm{maximize}}\,{P}_{s}^{(B,C)} & = & {q}_{1}{\alpha }_{1}{\alpha ^{\prime} }_{1}+{q}_{2}{\alpha }_{2}{\alpha ^{\prime} }_{2}\\ {\rm{subject}}\,{\rm{to}} &  & \mathrm{(1}-{\alpha }_{1}\mathrm{)(1}-{\alpha }_{2})-{s}^{2} > \mathrm{0,}\\  &  & \mathrm{(1}-{\alpha ^{\prime} }_{1}\mathrm{)(1}-{\alpha ^{\prime} }_{2})-{s^{\prime} }^{2}=0.\end{array}$$

First, let us consider $({\alpha ^{\prime} }_{1},{\alpha ^{\prime} }_{2})$. The objective function of Eq. (24) is a line with a slope of −*q*_1_*α*_1_/*q*_2_*α*_2_. $({\alpha ^{\prime} }_{1},{\alpha ^{\prime} }_{2})$, satisfying the objective function constraint. This is the tangential point between the equality condition and the line of objective function. At this point, the condition under which the objective function and the gradient become parallel is given by

25 $$\frac{\partial {P}_{s}^{(B,C)}/\partial {\alpha ^{\prime} }_{1}}{\partial {P}_{s}^{(B,C)}/\partial {\alpha ^{\prime} }_{2}}=\frac{{q}_{1}{\alpha }_{1}}{{q}_{2}{\alpha }_{2}}=\frac{1-{\alpha ^{\prime} }_{2}}{1-{\alpha ^{\prime} }_{1}}$$

Substituting Eq. (25) into the equality condition of Eq. (24), one can find $({\alpha ^{\prime} }_{1},{\alpha ^{\prime} }_{2})$ as follows:

26 $$\begin{array}{rcl}{\alpha ^{\prime} }_{1} & = & 1-s^{\prime} \sqrt{\frac{{q}_{2}{\alpha }_{2}}{{q}_{1}{\alpha }_{1}}},\\ {\alpha ^{\prime} }_{2} & = & 1-s^{\prime} \sqrt{\frac{{q}_{1}{\alpha }_{1}}{{q}_{2}{\alpha }_{2}}}.\end{array}$$

For $({\alpha ^{\prime} }_{1},{\alpha ^{\prime} }_{2})$ of Eq. (26), if ${\alpha ^{\prime} }_{1},{\alpha ^{\prime} }_{2} > 0$, the best strategy of Charlie is to discriminate two post-measurement states of Bob, which is the proof of Theorem 1.

Meanwhile, for $({\alpha ^{\prime} }_{1},{\alpha ^{\prime} }_{2})$, if one of ${\alpha ^{\prime} }_{1},{\alpha ^{\prime} }_{2} > 0$ is zero, the objective function of Eq. (24) becomes Eq. (11).

### **Proof of Theorem 2**.

Suppose that Bob and Charlie discriminate only one of three pure qutrit states of Alice. Eq. (4) is equivalent to the following optimization problem

27 $$\begin{array}{ll}{\rm{maximize}} & {P}_{s}^{(B,C)}={q}_{i}{\alpha }_{i}{\alpha ^{\prime} }_{i}\\ {\rm{subject}}\,{\rm{to}} & {\bar{\alpha }}_{i}-{s}_{i}^{2}{\bar{\alpha }}_{i}-{s}_{j}^{2}-{s}_{k}^{2}+2{s}_{i}{s}_{j}{s}_{k}\,\cos \,{\rm{\Phi }} > 0,\\  & {\bar{\alpha }^{\prime} }_{i}-{s^{\prime} }_{i}^{2}{\bar{\alpha }^{\prime} }_{i}-{s^{\prime} }_{j}^{2}-{s^{\prime} }_{k}^{2}+2{s^{\prime} }_{i}{s^{\prime} }_{j}{s^{\prime} }_{k}\,\cos \,{\rm{\Phi }}=0.\end{array}$$

where $${\bar{\alpha }}_{i}=1-{\alpha }_{i}$$, $${\bar{\alpha }^{\prime} }_{i}=1-{\alpha ^{\prime} }_{i}$$. (*α*_1_, *α*_2_, *α*_3_), satisfying Eq. (27), is a vertex of ∂*C*′. Substituting one of the vertices into Eq. (27), we can see that $${\alpha }_{i}=1-\sqrt{{\chi }_{i}({\rm{\Phi }})}$$ is the optimal condition, where $${\chi }_{i}({\rm{\Phi }})=({s}_{j}^{2}+{s}_{k}^{2}-$$$$2{s}_{i}{s}_{j}{s}_{k}\,\cos \,{\rm{\Phi }})/(1-{s}_{i}^{2})$$. Therefore, the optimized success probability becomes $${\rm{\max }}\,{P}_{s}^{(B,C)}={{\rm{\max }}}_{i}\{{q}_{i}\mathrm{(1}-{\chi }_{i}({\rm{\Phi }}))\}$$.

### **Proof of Theorem 3**.

Suppose that Bob and Charlie discriminate only two states out of three pure qutrit states of Alice. Eq. (4) can be written as the following optimization problem

28 $$\begin{array}{ll} & {\rm{maximize}}\,\,{s}^{(B,C)}={q}_{i}{\alpha }_{i}{\alpha }_{i^{\prime} }+{q}_{j}{\alpha }_{j}{\alpha }_{j^{\prime} }\\  & {\rm{subjectto}}\,\,\,\,{\bar{\alpha }}_{i}{\bar{\alpha }}_{j}-{s}_{i}^{2}{\bar{\alpha }}_{i}-{s}_{j}^{2}{\bar{\alpha }}_{j}-{s}_{k}^{2}+2{s}_{i}{s}_{j}{s}_{k}{\rm{c}}{\rm{o}}{\rm{s}}{\rm{\Phi }} > \mathrm{0,}\\  & \,\,\,\,\,\,\,\,\,\,\,\,\,\,\,\,\,\,\,\,{\bar{\alpha }}_{i}^{\prime} {\bar{\alpha }}_{j}^{\prime} -{s}_{i}^{2\prime} {\bar{\alpha }}_{i}^{\prime} -{s}_{j}^{2\prime} {\bar{\alpha }}_{j}^{\prime} -{s}_{k}^{2\prime} +2{s}_{i}^{\prime} {s}_{j}^{\prime} {s}_{k}^{\prime} {\rm{c}}{\rm{o}}{\rm{s}}{\rm{\Phi }}=0.\end{array}$$

$({\alpha ^{\prime} }_{1},{\alpha ^{\prime} }_{2})$, fulfilling the equality condition of Eq. (28), exists on the tangential point between a line $({q}_{i}{\alpha }_{i}){\alpha ^{\prime} }_{i}+({q}_{j}{\alpha }_{j}){\alpha ^{\prime} }_{j}$ and the equality condition. The condition for the tangential point to be located on ∂*C*′ becomes

29 $$\begin{array}{c}{\alpha }_{j} < \frac{{\alpha }_{i}{\mathrm{(1}-{\alpha }_{i}-{s}_{j}^{2})}^{2}}{{\alpha }_{i}{\mathrm{(1}-{\alpha }_{i}-{s}_{j}^{2})}^{2}+({q}_{j}/{q}_{i}){\xi }_{ij}{({\rm{\Phi }})}^{2}\mathrm{(1}-{\alpha }_{i})},\\ {\alpha }_{i} < \frac{{\alpha }_{j}{\mathrm{(1}-{\alpha }_{j}-{s}_{i}^{2})}^{2}}{{\alpha }_{j}{\mathrm{(1}-{\alpha }_{j}-{s}_{i}^{2})}^{2}+({q}_{i}/{q}_{j}){\xi }_{ij}{({\rm{\Phi }})}^{2}\mathrm{(1}-{\alpha }_{j})}.\end{array}$$

where ${\xi }_{ij}({\rm{\Phi }})=\sqrt{{s}_{i}^{2}{s}_{j}^{2}+{s}_{k}^{2}-2{s}_{i}{s}_{j}{s}_{k}\,\cos \,{\rm{\Phi }}}$. When (*α*_*i*_, *α*_*j*_) satisfies Eq. (29), the optimization problem is equivalent to Theorem 3.

### **Proof of Theorem 4**.

Suppose that Bob and Charlie discriminate every three pure qutrit state of Alice. The measurement conditions of Charlie for the best sequential state discrimination correspond to the tangential points between the plane $({q}_{1}{\alpha }_{1}){\alpha ^{\prime} }_{1}+({q}_{2}{\alpha }_{2}){\alpha ^{\prime} }_{2}+({q}_{3}{\alpha }_{3}){\alpha ^{\prime} }_{3}$ and $\partial C^{\prime}$. The tangential point can be analytically found when the geometric phase is Φ = 0, *π*^45^. However, for a general geometric phase, the condition for the plane to be tangent to ∂*C*′ is given by a 6-th order of algebraic equation^32,33^. For similarity, we assume that Φ = 0. Then, the tangential points become as follows:^32,33^.

30 $$\begin{array}{rcl}{\alpha ^{\prime} }_{1} & = & 1-\frac{{s^{\prime} }_{2}\sqrt{{q}_{3}{\alpha }_{3}}-{s^{\prime} }_{3}\sqrt{{q}_{2}{\alpha }_{2}}}{\sqrt{{q}_{1}{\alpha }_{1}}},\,{\alpha }_{2^{\prime} }=1-\frac{{s^{\prime} }_{1}\sqrt{{q}_{3}{\alpha }_{3}}-{s^{\prime} }_{3}\sqrt{{q}_{1}{\alpha }_{1}}}{\sqrt{{q}_{2}{\alpha }_{2}}},\\ {\alpha ^{\prime} }_{3} & = & 1-\frac{{s^{\prime} }_{2}\sqrt{{q}_{1}{\alpha }_{1}}+{s^{\prime} }_{1}\sqrt{{q}_{2}{\alpha }_{2}}}{\sqrt{{q}_{3}{\alpha }_{3}}}.\end{array}$$
